# Analyzing heterogeneity in biomarker discriminative performance through partial time-dependent receiver operating characteristic curve modeling

**DOI:** 10.1177/09622802241262521

**Published:** 2024-07-25

**Authors:** Xinyang Jiang, Wen Li, Kang Wang, Ruosha Li, Jing Ning

**Affiliations:** 1 Department of Biostatistics and Data Science, 12340 The University of Texas Health Science Center at Houston, Houston, TX, USA; 2 Department of Internal Medicine, The University of Texas Health Science Center at Houston McGovern Medical School, Houston, TX, USA; 3 Department of Biostatistics, The University of Texas MD Anderson Cancer Center, Houston, TX, USA

**Keywords:** Alzheimer, discriminative performance, partial AUC, pseudo partial-likelihood, time-dependent AUC

## Abstract

This study investigates the heterogeneity of a biomarker’s discriminative performance for predicting subsequent time-to-event outcomes across different patient subgroups. While the area under the curve (AUC) for the time-dependent receiver operating characteristic curve is commonly used to assess biomarker performance, the partial time-dependent AUC (PAUC) provides insights that are often more pertinent for population screening and diagnostic testing. To achieve this objective, we propose a regression model tailored for PAUC and develop two distinct estimation procedures for discrete and continuous covariates, employing a pseudo-partial likelihood method. Simulation studies are conducted to assess the performance of these procedures across various scenarios. We apply our model and inference procedure to the Alzheimer’s Disease Neuroimaging Initiative data set to evaluate potential heterogeneities in the discriminative performance of biomarkers for early Alzheimer’s disease diagnosis based on patients’ characteristics.

## 1. Introduction

Biomarkers have gained widespread use in biomedical research, encompassing various applications such as disease diagnosis, monitoring, and drug development.^1^ The discriminative ability of prognostic biomarkers, which are designed for identifying the likelihood of clinical events, disease recurrence, or progression in patients with a certain medical condition or disease, must undergo rigorous evaluation before their use in practice. In cases where research outcomes are time-to-events such as time-to-death, disease progression, or relapse,^2^ the conventional measures used for binary outcomes, including sensitivity, specificity, and the area under the curve (AUC) of the receiver operating characteristic (ROC) curve, have been extended to account for the dynamic nature of biomarker performance over time.^3–7^ The concept of time-dependent ROC and AUC in the context of both longitudinal and time-to-event outcomes has been extensively reviewed in Chapter 6 of “ROC Analysis for Classification and Prediction in Practice.”^8^ Additionally, joint modeling settings are also discussed in Chapter 7 of “Joint models for longitudinal and time-to-event data: With applications in R.”^9^

AUC and its time-dependent variants are commonly used as global measures to characterize the discriminative performance of biomarkers. One of their practical drawbacks is that it summarizes the entire ROC curve, although clinical and practical interests often focus on specific regions under the ROC curve rather than the full range of specificity. This is particularly relevant in cancer population screening, where high specificity is a top priority.^10^ Given the low incidence of cancer, it is preferable to prioritize high specificity to minimize the number of subjects who undergo unnecessary medical procedures and experience significant psychological stress.^11^ To address this clinical need, the partial area under the ROC curve (partial time-dependent area under the curve [PAUC]) has been recommended as a suitable metric for evaluating and comparing the performance of biomarkers, aligning with the clinical interest in targeted areas of the ROC curve.^12,13^

With the rapid advancements in biomarker research, it has become increasingly clear that identifying a single optimal biomarker with consistent discriminative performance across the entire population is not a realistic expectation. By analyzing the heterogeneity in biomarker discriminative performance, researchers can gain valuable insights into which specific groups or subpopulations exhibit favorable performance with the investigated biomarker, and conversely, which groups may not benefit as much. Understanding the limitations of biomarker performance in specific subgroups can guide further research and the development of improved biomarkers that may better serve those populations. For example, it has been reported that levels of prostate-specific antigen (PSA), a biomarker widely used to screen men for prostate cancer, tend to increase with age.^14^ With this finding, age-adjusted PSA would provide a more accurate screening tool for prostate cancer, particularly in older populations.

In the context of evaluating biomarker performance for binary outcomes, such as disease status, researchers have dedicated considerable attention to regression analysis to directly assess the discriminative performance using regression techniques. Particularly, several methods have been proposed for directly evaluating the performance of partial AUC using regression techniques with an emphasis on the control of higher specificity.^13,15,16^ When biomarkers are employed for risk stratification and monitoring disease progression, the outcomes of interest often involve time-to-event outcomes. In such circumstances, both nonparametric and regression approaches have been proposed to estimate the covariate-specific time-dependent ROC curve.^17–19^ To the best of our knowledge, no existing methods have been developed to model the time-dependent PAUC and investigate the heterogeneity in biomarker discriminative performance within the region of the clinically acceptable specificity over time. Therefore, our objective is to fill this research gap and gain insights into the variations in biomarker performance and its discriminatory abilities over different time periods.

One motivation study is to understand how the discriminative ability of biomarkers for Alzheimer’s disease (AD) is influenced by subjects’ characteristics. Data used in the preparation of this article were obtained from the AD Neuroimaging Initiative (ADNI) database (adni.loni.usc.edu). The ADNI was launched in 2003 as a public–private partnership, led by Principal Investigator Michael W. Weiner, MD. The primary goal of ADNI has been to test whether serial magnetic resonance imaging, positron emission tomography (PET), other biological markers, and clinical and neuropsychological assessment can be combined to measure the progression of mild cognitive impairment (MCI) and early AD.^20,21^ Among the factors being investigated, three cerebrospinal fluid (CSF) biomarkers, namely A β 42, total-tau (t-tau), and phosphorylated-tau (p-tau), have been identified as having the highest diagnostic potential.^22,23^ Furthermore, the existing literature suggests that the discriminative ability of biomarkers for the progression from MCI to AD may vary across different age groups.^18^ In light of these considerations, we aim to develop a robust statistical tool that rigorously evaluates the variability of a biomarker’s discriminative performance using the clinically meaningful measure known as PAUC.

The remaining sections of this article are organized as follows. In Section 2, we begin by providing a definition of the covariate-specific PAUC. We then introduce the regression model for the PAUC and propose a pseudo-partial likelihood approach to estimate the model parameters. Initially, we focus on categorical covariates and subsequently extend the estimation procedure to handle continuous covariates. Furthermore, we establish the asymptotic behavior of the proposed approach. In Section 3, we conduct comprehensive simulation studies to assess the finite sample performance of the proposed methods under various scenarios. In Section 4, we apply the proposed method to the ADNI data set and evaluate how the discriminative performance of biomarkers, as characterized by the PAUC, changes with different subjects’ characteristics. We conclude the article with a discussion of our findings in Section 5.

## 2. Methods

### 2.1. Notations and time-dependent measures

Denote the event time as $\tilde{T}$ and the censoring time as C , where C is assumed to be independent of $\tilde{T}$ . The observed survival time is defined as $T=\min(\tilde{T},C)$ , and the event indicator is $\delta =I(\tilde{T}\leq C)$ , where I(.) denotes the indicator function. The time-invariant biomarker is denoted as Y , with higher values indicating a higher risk of disease. The vector of covariates is represented by X .

Building upon the framework proposed by Heagerty and Zheng,^3^ we adopt the incident/dynamic definition of time-dependent accuracy summaries to incorporate covariate information. Specifically, the covariate-specific incident sensitivity, dynamic false positive (i.e. 1-specificity), and the corresponding time-dependent ROC are defined as follows:

$$\begin{array}{rl} {\mathrm{TP}}_{t}(y|x) & =P(Y>y|\tilde{T}=t,X=x)\\ {\mathrm{FP}}_{t}(y|x) & =P(Y>y|\tilde{T}>t,X=x) \end{array}$$

and

$${\mathrm{ROC}}_{t}(p|x)={\mathrm{TP}}_{t}\{{\mathrm{FP}}_{t}^{-1}(p|X=x)\},\;p\in [0,1]$$

To focus on regions of p that hold particular relevance for practical applications and exclude regions that are frequently not of interest, we consider the PAUC, which summarizes the discriminative performance of the biomarker in a more targeted and meaningful manner. Specifically, by incorporating time and covariate information for the partial AUC definition in Dodd and Pepe,^13^ we define the covariate-specific and time-varying PAUC as follows:

$$\begin{array}{rl} \mathrm{PAUC}(t,u;x) & =\int_{0}^{u}{\mathrm{ROC}}_{t}(p|x)dp\\ & =P(Y_{1}>Y_{2},Y_{2}\in \{{\mathrm{FP}}_{t}^{-1}(u|x),{\mathrm{FP}}_{t}^{-1}(0|x)\}|{\tilde{T}}_{1}=t,{\tilde{T}}_{2}>t,X_{1}=X_{2}=x) \end{array}$$

In this definition, $X_{1}$ represents the covariate information for the case (i.e. ${\tilde{T}}_{1}=t$ ), while $X_{2}$ represents the covariate information for the control (i.e. ${\tilde{T}}_{2}>t$ ). Note that we focus on subjects with the same covariate information, following the recommendation in the literature,^15^ to generalize covariate-specific measures. To account for the influence of u on the scale of the PAUC(t;u,x,θ) , we consider a standardized PAUC^13^ as follows:

(1) $${\mathrm{PAUC}}^{*}(t;u,x)=\mathrm{PAUC}(t;u,x)/u$$

We denote the range of biomarker values based on the constraint of the dynamic false positive rate as $\left[{\mathrm{FP}}_{t}^{-1}(u|x),{\mathrm{FP}}_{t}^{-1}(0|x)\right]$ , represented as $B_{t}(u|x)$ .

### 2.2. Model and estimation procedures

#### 2.2.1. Model for the covariate-specific PAUC

Motivated by the need to comprehend the heterogeneity of biomarker discriminative performance, we propose a regression model for the covariate-specific PAUC. Using a link function η(.) , fractional polynomials of t with coefficients α , and coefficients β for x , we formulate the covariate-specific PAUC as follows:

(2) $$\eta \{{\mathrm{PAUC}}^{*}(t;u,x,\theta )\}=\sum_{k=0}^{K}\alpha_{k}t^{\left(p_{k}\right)}+x^{T}\beta$$

where $\theta^{T}$ represents the vector of coefficients $\left(\alpha^{T},\beta^{T}\right)$ , and

$$\{t^{\left(p_{k}\right)},k=0,\ldots ,7\}=\left\{1,t^{-2},t^{-1},t^{-1/2},\log\;(t),t^{1/2},t,t^{2}\right\}$$

The model in equation (2) implies the baseline PAUC when all covariates are set to 0 is

$${\mathrm{PAUC}}_{0}^{*}(t;u,\alpha )=\eta^{-1}\left\{\alpha_{0}+\alpha_{1}t^{-2}+\alpha_{2}t^{-1}+\alpha_{3}t^{-1/2}+\alpha_{4}\log\;(t)+\alpha_{5}t^{1/2}+\alpha_{6}t+\alpha_{7}t^{2}\right\}$$

When employing the logit link function η(⋅) , the exponentiation of the regression coefficient represents the odds ratio of the corresponding covariate on the partial AUC. In simpler terms, the coefficient can be interpreted as the expected change in log odds of the partial AUC per unit change in its associated covariate for continuous covariates. To exemplify, following Dodd and Pepe’s work,^13^ we consider a simple model featuring one covariate, where the log odds of covariate-specific standardized PAUC are defined as follows:

$$\log\;\left\{\frac{{\mathrm{PAUC}}^{*}(t;x)}{1{\mathrm{PAUC}}^{*}(t;x)}\right\}=\log\;\left\{\frac{\mathrm{PAUC}(t;x)}{u\mathrm{PAUC}(t;x)}\right\}=\sum_{k=0}^{K}\alpha_{k}t^{\left(p_{k}\right)}+\beta_{1}x$$

Within this model, if X is a continuous variable, then for each one-unit increase in X , the term $\beta_{1}$ describes the corresponding change in the odds of the covariate-specific PAUC* at the log scale. If X is a binary variable such as gender (with female coded as 0 and male as 1), $e^{\beta_{1}}$ indicates the odds ratio of the covariate-specific PAUC* when comparing males to females. Furthermore, if $e^{\beta_{1}}>1$ , it suggests that the biomarker shows better discrimination capability for males.

Acknowledging clinical interest in the discriminative accuracy evaluation of a biomarker, which often lies in the discrimination between cases and controls within the same subgroup,^15^ we have enforced the use of identical covariate information for both cases and controls in our regression model. Although this model constraint is clinically meaningful, it presents a new challenge in estimating regression coefficients, particularly for continuous covariates due to the inherent curse of dimensionality.^24^ In the following sections, we present separate estimation procedures tailored for handling discrete and continuous covariates. These procedures are designed to effectively tackle the challenges introduced by the constraint of using identical covariate information for cases and controls in our regression model and also incorporate covariate distance between cases and controls to enable information borrowing.

#### 2.2.2. Estimation procedure for discrete covariates

For a given subject i with event time $T_{i}=t_{i}$ and covariate vector $X_{i}=x_{i}$ , we define a covariate-specific risk set as follows:

$$R\left(t_{i},x_{i}\right)=\left\{j:T_{j}>t_{i},X_{j}=x_{i}\right\}$$

which is a generalization of the conventional risk set. The covariate-specific risk set incorporates specific covariate information into the risk set definition, by restricting subjects with the same covariate information to be grouped together for the classification of cases and controls. Denote the number of individuals at risk at time t with covariate x as n(t,x) , where $n(t,x)=\sum_{j=1}^{n}I(T_{j}>t,X_{j}=x)$ . For subject i with an observed failure time $T_{i}=t_{i}$ and covariate $X_{i}=x_{i}$ , we focus on a conditional event within the risk set $R(t_{i},x_{i})$ and the predefined range for the biomarker, introduced by the dynamic false positive rate constraint:

(3) $$e_{ij}=I\left\{Y_{i}>Y_{j}|\delta_{i}=1,j\in R(t_{i},x_{i}),Y_{j}\in B_{t_{i}}(u|x_{i})\right\}$$

Given a cutoff value c , the covariate-specific dynamic false positive rate within the aforementioned risk set can be calculated non-parametrically as:

$${\mathrm{FP}}_{t_{i}}(c|x_{i})=\frac{\sum_{j\in R(t_{i},x_{i})}I(Y_{j}>c)}{n(t_{i},x_{i})}$$

which allows for straightforward verification of the condition $Y_{j}\in B_{t_{i}}(u|x_{i})$ . Under the assumption that a higher value of the biomarker indicates an increased risk of disease, we refer to the event $e_{ij}$ as a concordant event, as subject i experienced the event earlier and had a higher value of the biomarker compared to subject j . The event $e_{ij}$ is closely connected to the standardized PAUC and, in fact, follows a Bernoulli distribution with a probability of ${\mathrm{PAUC}}^{*}(t_{i};u,x_{i},\theta )$ . This connection serves as the foundation for building the likelihood to estimate parameter θ . Specifically, we multiply the probabilities of all such events across all risk sets constructed from the observed data. This gives rise to the following pseudo-partial likelihood:

(4) $$L(\theta )\propto \prod_{i=1}^{n}{\left(\prod_{j\in R(t_{i},x_{i})}{\left[{\mathrm{PAUC}}^{*}(t_{i};u,x_{i},\theta {)}^{e_{ij}}{\left\{1-{\mathrm{PAUC}}^{*}\left(t_{i};u,x_{i},\theta \right)\right\}}^{\left(1-e_{ij}\right)}\right]}^{I\{Y_{j}\in B_{t_{i}}(u|x_{i})\}}\right)}^{\delta_{i}}$$

#### 2.2.3. Estimation procedure for continuous covariates

When dealing with continuous covariates, the strategy of grouping subjects with the same covariate information to define the at-risk set is not feasible. Alternatively, we propose to assign weights to the controls, who are event-free at the event time of a case, based on the distance in covariates between the case and its controls, using a kernel smoothing technique. For illustrative purposes, we consider the scenario with one continuous covariate. Under this approach, we utilize the conventional risk set, defined as $R(t_{i})=\{j:T_{j}>t_{i}\}$ . However, we incorporate covariate information to define a weighted number of subjects in the risk set: $n_{w}(t_{i},x_{i})=\sum_{j=1}^{n}I(T_{j}>t_{i})K_{h}(x_{i},X_{j})$ , where $K_{h}(x_{i},X_{j})=K\left\{(x_{i}-X_{j})/h\right\}/h$ represents a kernel function with a bandwidth of h .^24^ Under the subsequent simulations and data application, we use the Epanechnikov kernel function $K(x)=0.75(1-x^{2})I(|x|<1)$ .^25^ We select the bandwidth as $h=range(x)\times (u\times n{)}^{-1/3}$ , recommended by Hu et al.^24^

Accordingly, the false positive rate would be estimated by weighting the controls based on their covariate information in the risk set:

(5) $${\mathrm{FP}}_{t_{i}}^{w}(c|x_{i})=\frac{\sum_{j\in R(t_{i})}I(Y_{j}>c)K_{h}(x_{i},X_{j})}{n_{w}(t_{i},x_{i})}$$

We next consider two conditional events in the risk set and the predefined range for the biomarker by the dynamic false positive rate constraint, termed as kernel-based concordant and discordant events

(6) $$\begin{array}{rl} e_{ij}^{c} & =K_{h}(x_{i},X_{j})I\left\{Y_{i}>Y_{j}|T_{i}=t_{i},\delta_{i}=1,j\in R(t_{i}),Y_{j}\in B_{t_{i}}^{w}(u|x_{i})\right\},\text{ and}\\ e_{ij}^{d} & =K_{h}(x_{i},X_{j})I\left\{Y_{i}\leq Y_{j}|T_{i}=t_{i},\delta_{i}=1,j\in R(t_{i}),Y_{j}\in B_{t_{i}}^{w}(u|x_{i})\right\} \end{array}$$

where $B_{t_{i}}^{w}(u|x_{i})$ denotes the biomarker range based on the weighted dynamic false positive rate. Notably, the concordant or discordant counts for pairs with similar covariate information have larger weights compared to pairs with more different covariate values. Furthermore, we construct a weighted pseudo-partial likelihood for continuous covariates as follows:

(7) $$L(\theta )\propto \prod_{i=1}^{n}{\left(\prod_{j\in R(t_{i})}{\left[{\mathrm{PAUC}}^{*}(t_{i};u,x_{i},\theta {)}^{e_{ij}^{c}}{\left\{1-{\mathrm{PAUC}}^{*}\left(t_{i};u,x_{i},\theta \right)\right\}}^{e_{ij}^{d}}\right]}^{I\{Y_{j}\in B_{t_{i}}^{w}(u|x_{i})\}}\right)}^{\delta_{i}}$$

Note that the univariate kernel can be extended to a multivariate form, given by $K_{h}({\text{x}}_{i},{\text{X}}_{j})=K\{||{\text{x}}_{i}-{\text{X}}_{j}||/h\}/h$ . This generalization allows for the handling of multiple continuous covariates, where ||.|| represents the Euclidean norm. In scenarios involving both discrete and continuous covariates, we can integrate the aforementioned estimation procedures. The discrete covariates play a role in determining the composition of risk sets, while the continuous covariates influence the weights of individual members within these risk sets.

Our proposed pseudo-partial likelihood is inspired by the pseudo-likelihood, which has been demonstrated as the most effective approach for analyzing censored data within the framework of the Cox proportional hazards model. To illustrate our method, we consider a scenario with discrete covariates. At each observed failure time point, denoted as t , we construct a covariate-specific risk set. This risk set comprises both censored and uncensored observations, encompassing individuals with times exceeding t and sharing the same covariate value. We then compare the biomarker values between the subject failing at time t and all subjects within the risk set, including censored subjects. With this information, we derive the pseudo partial function as defined in equation (4). By adopting this approach, censored subjects can contribute to multiple risk sets, thus enhancing the overall likelihood estimation.

### 2.3. Implementation

In terms of implementation, while developing computational codes to maximize our likelihood functions should not be complex, leveraging existing software would facilitate broader adoption of our proposed methods. Recognizing the similarity between the pseudo-partial likelihood functions presented in (4) and (7) and the likelihood function commonly associated with binary outcomes, we reshape the data and leverage the existing software for fitting generalized linear model for estimation.^26^ Algorithm 1 summarizes the implementation procedure with one discrete (e.g. $X_{d}$ ) and one continuous covariate (e.g. $X_{c}$ ), which can be easily extended to cases with multiple discrete and continuous covariates. A pivotal step involves contracting n(t,x) binary outcomes at each risk set based on concordant or discordant statuses between the subject failing at time t and all subjects within the risk set, as outlined in Algorithm 1. Once more, censored subjects are included in risk sets, and their information is leveraged for constructing these binary outcomes.

**Table:**
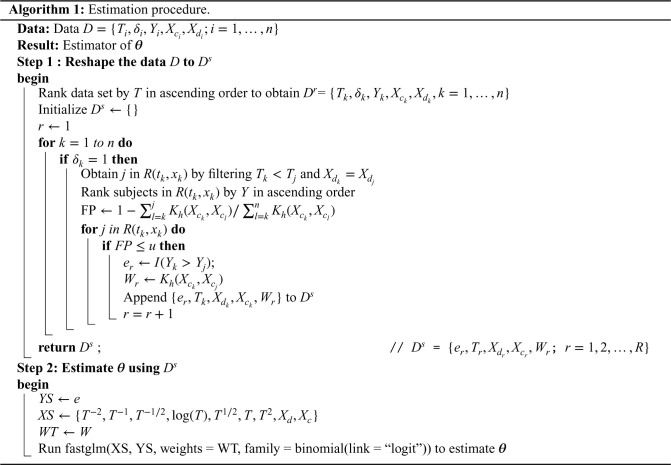


### 2.4. Asymptotic properties

In this section, we delve into the asymptotic properties of the proposed method. Note that the concordant events or the kernel-based concordant events do not exhibit mutual independence. As a result, the likelihood formulated using these events cannot be readily treated as a conventional likelihood for inference purposes, and overcoming this challenge requires additional considerations. Without loss of generality, we assume the model includes one discrete covariate $X_{d}$ and one continuous covariate $X_{c}$ . The log-likelihood for this scenario can be expressed as follows:

(8) $$\begin{array}{rl} l(\theta ) & =\sum_{i=1}^{n}\sum_{j=1}^{n}\delta_{i}I\{T_{j}>T_{i},X_{di}=X_{dj},Y_{j}\in B_{T_{i}}^{w}(u|X_{i})\}K_{h}(X_{ci},X_{cj})\\ & \times \left[I(Y_{i}>Y_{j})\log\;\left\{{\mathrm{PAUC}}^{*}(T_{i},X_{i};\theta )\right\}+I(Y_{i}\leq Y_{j})\log\;\left\{(1{\mathrm{PAUC}}^{*}(T_{i},X_{i};\theta )\right\}\right] \end{array}$$

Taking the first derivative of equation (8), we have the following score equation:

(9) $$\begin{array}{rl} S(\theta ) & =\sum_{i=1}^{n}\sum_{j=1}^{n}\delta_{i}I\{T_{j}>T_{i},X_{di}=X_{dj},Y_{j}\in B_{T_{i}}^{w}(u|X_{i})\}K_{h}(X_{ci},X_{cj})\\ & \;\times \left\{I(Y_{i}>Y_{j})\frac{\nabla_{\theta }{\mathrm{PAUC}}^{*}(T_{i},X_{i};\theta )}{{\mathrm{PAUC}}^{*}(T_{i},X_{i};\theta )}-I(Y_{i}\leq Y_{j})\frac{\nabla_{\theta }{\mathrm{PAUC}}^{*}(T_{i},X_{i};\theta )}{1-{\mathrm{PAUC}}^{*}(T_{i},X_{i};\theta )}\right\} \end{array}$$

where $\nabla_{\theta }f(\theta )$ is the first derivative of the function f(θ) with respect to θ . For simplification, denote $S(\theta )=\sum_{i=1}^{n}\sum_{j=1}^{n}S_{ij}(\theta )$ . Let $\hat{\theta }$ be the solution of S(θ)=0 , and $\theta_{0}$ be the true value of θ . To establish the link between the score equation and a U-statistics with a degree of 2, we construct a symmetric kernel function by $G_{ij}(\theta )=\{S_{ij}(\theta )+S_{ji}(\theta )\}/2.$ Subject to regularity conditions outlined in the supplemental materials and employing the Taylor expansion and projection theorem for U-statistics, we can establish the asymptotic properties of the estimator $\hat{\theta }$ , which are summarized in the following theorem.

Theorem 1 Under the regularity conditions (A1–A6) in the supplemental materials, the estimator $\hat{\theta }$ is consistent and asymptotically follows a normal distribution with a mean of $\theta_{0}$ and a covariance matrix of $V=\Sigma_{1}^{-1}\Sigma_{2}\Sigma_{1}-1$ , where $\Sigma_{1}=E\{-\partial S_{12}(\theta )/\partial \theta \}{|}_{\theta =\theta_{0}}$ and $\Sigma_{2}=4cov\{G_{12}(\theta ),G_{13}(\theta )\}{|}_{\theta =\theta_{0}}$ .

The covariance matrix V can be consistently estimated by plugging in $\hat{\theta }$ and empirical counterparts of $\Sigma_{1}$ and $\Sigma_{2}$ . Leveraging the asymptotic normality of $\hat{\theta }$ , we apply the multivariate delta method to derive the asymptotic distribution of ${\mathrm{PAUC}}^{*}(T_{i},X;\hat{\theta })$ for any fixed x and t , which are summarized in the following corollary.

Corollary 1.1 Under the regularity conditions (A1–A6) in the supplemental materials, ${PAUC}^{*}(t;u,x,\hat{\theta })$ is a consistent estimator of ${PAUC}^{*}(t;u,x,\theta_{0})$ , and $\sqrt{n}({PAUC}^{*}(t;u,x,\hat{\theta })-{PAUC}^{*}(t;u,x,\theta_{0}))$ converges to a normal distribution with a mean of 0 and a variance of $\{\nabla_{\theta }\eta (\theta_{0}^{T}c){\}}^{-1}V\{\nabla_{\theta }\eta (\theta_{0}^{T}c){\}}^{-1}$ , where c denotes the vector $(1,t^{-2},t^{-1},t^{-1/2},\log\;(t),t^{1/2},t,t^{2},x^{T}{)}^{T}$ .

## 3. Simulation

For the purpose of evaluating the performance of our proposed methods, we conducted a comprehensive simulation study under various data-generating scenarios.

### 3.1. Simulation settings

The biomarker Y and the log of survival time $\tilde{T}$ were generated from a bivariate normal distribution with a variance of 1 and a correlation of ρ=−0.7 . The choice of a negative correlation reflects the assumption that higher biomarker values correspond to a greater indication of disease risk. The censoring time was generated independently from a uniform distribution U(0,τ) , where τ was chosen to provide the desirable censoring rate ( 15% or 30% ). The false positive rate was controlled at u=0.2,0.4,0.6,0.8,1 . For each setting, sample sizes of n=500 and n=1000 were used. The true values of all regression coefficients were approximated by using a large data set with a sample size of n=30,000 .

We considered three scenarios to evaluate the performance of the proposed methods when there are different types of covariates. In Scenario 1, we evaluated the performance of the proposed methods in the presence of a discrete covariate $X_{d}$ , which was generated using a Bernoulli distribution with a probability of 0.5 . Subsequently, the means of the biomarker and survival time were $\mu_{Y}=-X_{d}-1$ and $\mu_{\log\;(\tilde{T})}=X_{d}+1$ . The model used for ${\mathrm{PAUC}}^{*}(t;x_{c},\alpha ,\beta )$ under this scenario was assumed to take the form:

$$\mathrm{logit}\{{\mathrm{PAUC}}^{*}(t;x_{d})\}=\alpha_{0}+\alpha_{1}t^{-2}+\alpha_{2}t^{-1}+\alpha_{3}t^{-1/2}+\alpha_{4}\log\;(t)+\alpha_{5}t^{1/2}+\alpha_{6}t+\alpha_{7}t^{2}+\beta_{d}x_{d}$$

The focus of Scenario 2 is to evaluate the method’s performance with a continuous covariate $X_{c}$ , where $X_{c}$ was generated from a uniform distribution within the range of 0 to 1 . The mean of the biomarker was set to $\mu_{Y}=-X_{c}-1$ , while the mean of the log of survival time was set to $\mu_{\log\;(\tilde{T})}=X_{c}+1$ . The model used for this scenario was formulated as follows:

$$\mathrm{logit}\{{\mathrm{PAUC}}^{*}(t;x_{c})\}=\alpha_{0}+\alpha_{1}t^{-2}+\alpha_{2}t^{-1}+\alpha_{3}t^{-1/2}+\alpha_{4}\log\;(t)+\alpha_{5}t^{1/2}+\alpha_{6}t+\alpha_{7}t^{2}+\beta_{c}x_{c}$$

In Scenario 3, two covariates were generated independently: $X_{d}∼\mathrm{Bernoulli}(0.5)$ and $X_{c}∼\mathrm{Uniform}(0,1)$ . The mean of the biomarker and the time were set to $\mu_{Y}=-X_{d}-X_{c}-1$ and $\mu_{\log\;(\tilde{T})}=X_{d}+X_{c}+1$ . Accordingly, the model was specified as follows:

$$\begin{array}{rl} \mathrm{logit}\{{\mathrm{PAUC}}^{*}(t;x_{d},x_{c})\} & =\alpha_{0}+\alpha_{1}t^{-2}+\alpha_{2}t^{-1}+\alpha_{3}t^{-1/2}+\alpha_{4}\log\;(t)+\alpha_{5}t^{1/2}+\alpha_{6}t+\alpha_{7}t^{2}+\beta_{d}x_{d}+\beta_{c}x_{c} \end{array}$$

### 3.2. Simulation results

All results were derived from 1000 simulated data sets. Due to space constraints, we have included simulation results for Scenario 3 within the main body of the paper and left simulation results for Scenarios 1 and 2 in the supplemental materials.

For Scenarios 1 and 2, empirical biases (Bias) and standard deviations (SDs) of the proposed estimators under various sample sizes (500 or 1000), censoring rates ( 0% , 15% , or 30% ), and threshold values ( u ) for the PAUC are displaced in Figures S1 and S2 of the supplemental materials. These figures showcase the satisfactory performance of the estimators, revealing only a minor increase in bias under high-censoring rates or with small-threshold values. It is noteworthy that the effective sample sizes significantly depend on the PAUC’s threshold value, given a specific sample size. For smaller threshold values, larger sample sizes are needed to achieve reliable estimator performance.

The empirical coverage probabilities (CPs) of 95% confidence intervals under Scenarios 1 and 2 are summarized in Figures S1 and S2 of the supplemental materials. Despite occasional underestimation of standard error (SE) when compared to the empirical SD, the CPs consistently remained in close proximity to the nominal level. Graphical representations of estimation and confidence intervals for baseline PAUC are displaced in Figures S3 and S4. These visuals effectively illustrated the small biases of estimators to the true value, particularly with larger sample sizes. Variance escalation over time, attributed to the diminishing risk set size, was also evident. As expected, the overall performance notably improves with an increased sample size of 800 , manifesting in reduced bias, variance, and coverage probabilities approaching the nominal level.

**Figure 1.**
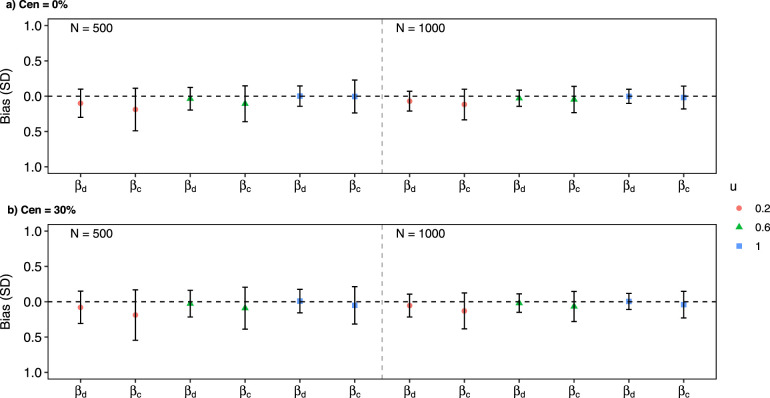
Simulation results: biases and empirical standard deviations in scenario 3.

**Figure 2.**
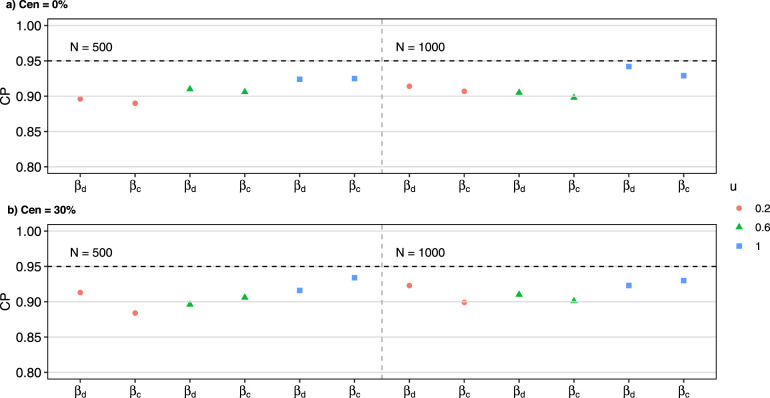
Simulation results: empirical coverage probabilities in scenario 3.

**Figure 3.**
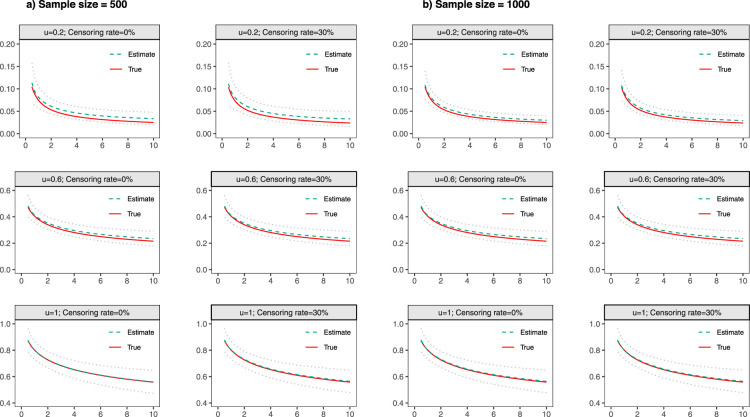
Estimated baseline ${\mathrm{PAUC}}_{0}(t,\alpha )$ with 95% confidence intervals in scenario 3.

Detailed simulation results under Scenario 3 are presented in Figures 1 to 3. To elaborate, Figure 1 provides an overview of biases and SDs for estimated regression coefficients. Figure 2 illustrates the CPs of these estimates. Additionally, Figure 3 displays the average of the estimated baseline time-dependent partial AUC, along with their corresponding 95% empirical confidence intervals.

With both continuous and discrete covariates involved in the model, the simulation results had a minimal bias, with slightly larger biases observed under smaller effective sample sizes (i.e. smaller values of u and n , or larger censoring rates) for estimation and inference. This occasionally resulted in the underestimation of variance by using the asymptotic formula. Consequently, the CPs were marginally lower than the nominal value, although still within a reasonable range. The variance estimation bias diminished with larger sample sizes, reduced censoring rates, or larger threshold values for the PAUC, thereby yielding satisfactory CPs. Moreover, the estimation results for baseline partial AUC ( ${\mathrm{PAUC}}_{0}$ ) remain virtually unbiased. Consistent with previous scenarios, we observed slightly increased variance under higher censoring rates or smaller PAUC threshold values. It is worth noting that in our simulation settings, ${\mathrm{PAUC}}_{0}(t)$ values exhibit higher initial values followed by a decreasing trend over time, indicative of the biomarker’s strong short-term discriminative capability and under-optimal discriminative performance over time. For a detailed breakdown of numerical values associated with the figures, please refer to Tables S1–S3 in the supplemental materials.

Additional simulation studies have been conducted to further assess the performance of our proposed method. These studies include evaluations of models with varying numbers of polynomials included, models incorporating interaction terms, and additional settings with censoring times derived from the ADNI data. Detailed descriptions of these additional simulations and their results can be found in Section S2 of the supplemental materials.

## 4. Real data application

In recent studies, three CSF biomarkers—A β 42, t-tau, and p-tau—have gained prominence as effective indicators for the early detection of AD. In this context, we applied the proposed model and inference procedure to the ADNI database to investigate the impact of subjects’ characteristics on the performance of these biomarkers. This analysis aims to evaluate potential heterogeneity in biomarker performance and identify subpopulations that may exhibit unfavorable performance with the investigated biomarkers, thereby highlighting the need for improved biomarkers.

During the ADNI study, participants were enrolled with varying AD statuses, including normal aging, early mild cognitive impairment, and late mild cognitive impairment or AD. These participants were prospectively followed and reassessed over time to track the progression of the disease. The primary outcome of our investigation was the time to diagnosis of AD. Our analytic cohort comprised 957 subjects after excluding prevalent cases (subjects with AD before study enrollment). The median follow-up time was 48 months, with a range from 6 to 180 months. Among these subjects, 261 (27.3%) were diagnosed with AD or died during the study period. The analytical cohort comprised 434 female patients (45.4%) and 523 male patients (54.6%). The median patient age was 73 years, with an interquartile range of 66 to 78 years (Table S7 in supplemental materials).

We fitted three separate regression models to evaluate whether the discriminative ability of the three aforementioned biomarkers for AD progression would be influenced by subjects’ age and gender. In these regression models, age was treated as a continuous variable centered at 75 years old, and the reference group for gender was set as male. Previous research by Pettigrew et al.^27^ suggests that age may exhibit a nonlinear effect on AD progression, prompting the inclusion of a quadratic term in the regression model. Following this precedent, we incorporated the quadratic term in our model. Fitting results affirmed that a straightforward linear effect of age might not sufficiently capture the nuances of age-related effects. Let $X_{d}$ represent gender (female = 1), and $X_{c}$ represent age, where $X_{c}-75$ represents age centered at 75 years. The model is specified as follows:

$$\begin{array}{rl} \mathrm{logit}\{{\mathrm{PAUC}}^{*}(t;x_{d},x_{c})\} & =\alpha_{0}+\alpha_{1}t^{-2}+\alpha_{2}t^{-1}+\alpha_{3}t^{-1/2}+\alpha_{4}\log\;(t)\\ & \;+\alpha_{5}t^{1/2}+\alpha_{6}t+\alpha_{7}t^{2}+\beta_{1}x_{d}+\beta_{2}(x_{c}-75)/10+\beta_{3}\{(x_{c}-75)/10{\}}^{2} \end{array}$$

For u=0.2 , we have simplified the model specifications by employing three fractional polynomial terms $t^{1/2}$ , t , and $t^{2}$ .

Table 1 summarizes the estimated regression coefficients with different threshold values on the PAUC for the biomarker t-tau, while Tables S4 and S5 of the supplemental materials list the estimated regression coefficients for the other two biomarkers. For the t-tau biomarker, the influence of subjects’ age on their performance was contingent on the chosen threshold values. For values of u below or equal to 0.6, age did not yield a statistically significant impact. However, as the threshold increased, age began to play a discernible role in affecting the t -tau biomarker’s performance. For instance, when u=1 , each additional year of age in patients aged 75 was associated with an odds ratio of 0.969. This suggests that the t-tau biomarker exhibits superior discriminatory capacity in younger patients. To determine the odds ratio for a one-year increase in age at the age of 75 after controlling for gender, we inserted the values into our model as follows:

$$\frac{\exp\{\beta_{2}(76-75)/10+\beta_{3}\{(76-75)/10{\}}^{2}\}}{\exp\{\beta_{2}(75-75)/10+\beta_{3}\{(75-75)/10{\}}^{2}\}}=\exp\;\{0.1\beta_{2}+0.1^{2}\beta_{3}\}$$

Meanwhile, the discriminatory power of t-tau was found to be more pronounced in female patients compared to male patients. Specifically, when comparing female to male subjects, the PAUC odds ratios were 1.586 ( p -value = 0.056), 1.564 ( p -value = 0.017), and 1.582 ( p -value = 0.009) for u=0.2 , 0.8 , and 1 , respectively. We observed similar findings for the other two biomarkers and omitted detailed results here.

**Table 1.** Estimated covariate effects on the PAUC of biomarker t -tau using the ADNI data.

u	Covariate	Estimate	SE	Wald	P value
0.2	Gender (female)	0.461	0.241	1.914	0.056
	Age	0.038	0.226	0.170	0.865
	Age^2^	−0.237	0.249	−0.952	0.341
0.4	Gender (female)	0.391	0.210	1.862	0.063
	Age	−0.116	0.191	−0.605	0.545
	Age^2^	−0.210	0.199	−1.058	0.290
0.6	Gender (female)	0.380	0.198	1.923	0.055
	Age	−0.258	0.184	−1.401	0.161
	Age^2^	−0.346	0.189	−1.830	0.067
0.8	Gender (female)	0.447	0.187	2.388	0.017
	Age	−0.228	0.172	−1.328	0.184
	Age^2^	−0.381	0.176	−2.165	0.030
1	Gender (female)	0.459	0.175	2.626	0.009
	Age	−0.280	0.159	−1.764	0.078
	Age^2^	−0.352	0.161	−2.189	0.029

PAUC: partial time-dependent area under the curve; ADNI: Alzheimer’s disease neuroimaging initiative; SE: standard error.

**Figure 4.**
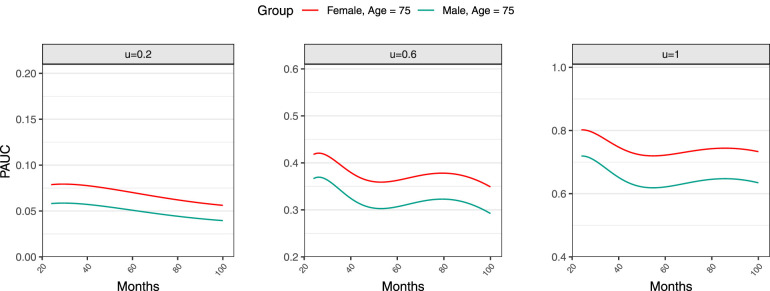
Estimated time-dependent time-dependent area under the curve (PAUC) curves by sex.

For a visual representation, Figure 4 plots the PAUC across different subgroups determined by subjects’ gender and age. The estimated ${\text{PAUC}}^{*}(t;u,x,\hat{\theta })$ function exhibited an overall descending trend over time, although the patterns were not strictly monotonic.

## 5. Discussion

Recognizing the clinical and practical interest in specific regions under the ROC curve, we introduce the PAUC regression model and inference procedure to comprehend the heterogeneity in the discriminative performance of biomarkers with time-to-event outcomes. One notable advantage of our proposed estimation approach lies in its simplicity for handling right censoring, obviating the need for an inverse weighting approach that involves estimating the censoring distribution. Furthermore, the analytical formula provided for variance estimation alleviates computational burden in practical applications.

While our primary focus in this paper is the assessment of heterogeneity in the discriminative performance of individual biomarkers, the proposed model and inference procedure can be extended to evaluate the performance of biomarker combinations or risk stratification scores. This extension would require the incorporation of cross-validation procedures to address additional variability when coefficients for biomarker combinations or risk stratification scores are unknown and need to be estimated. While our work does not focus specifically on formal heterogeneity testing, our proposed regression models can indeed serve as valuable tools for assessing the heterogeneity of a biomarker’s effectiveness across various subgroups. By evaluating regression coefficients associated with covariates, such as age and gender, our models enable us to evaluate whether there are significant differences in the discriminative performance of the biomarker across diverse subgroups. Specifically, we can employ Wald-type tests to determine whether regression coefficients associated with these covariates are significantly different from zero. These tests allow us to investigate whether there are notable variations in the biomarker’s discriminative performance among different subgroups. Identifying such disparities can be instrumental in pinpointing specific subgroups where further improvement in biomarker effectiveness may be necessary.

One assumption underlying our proposed inference procedure is the assumption of independent censoring. It is important to acknowledge that in the presence of competing risks, this assumption might be violated. However, the proposed model and inference procedure can be generalized to accommodate competing risks by drawing on the existing literature on time-dependent predictive measures.^28^ This, however, falls beyond the scope of this present paper. Another challenge in our method is assessing model fitting for the PAUC. Standard tools, such as residual plots, are not directly applicable toevaluating the adequacy of the model. Developing rigorous statistical tools for this purpose is worthy of future research.

## Supplemental Material

sj-pdf-1-smm-10.1177_09622802241262521 - Supplemental material for Analyzing heterogeneity in biomarker discriminative performance through partial time-dependent receiver operating characteristic curve modeling Supplemental material, sj-pdf-1-smm-10.1177_09622802241262521 for Analyzing heterogeneity in biomarker discriminative performance through partial time-dependent receiver operating characteristic curve modeling by Xinyang Jiang, Wen Li, Kang Wang, Ruosha Li and Jing Ning in Statistical Methods in Medical Research
