# Supplementary material for: Analyzing heterogeneity in biomarker discriminative performance through partial time-dependent receiver operating characteristic curve modeling
Source: Stat Methods Med Res. 2024 Jul 25;33(8):1424–36. doi: 10.1177/09622802241262521 (PMC11449645; doi:10.1177/09622802241262521)
Supplement: sj-pdf-1-smm-10.1177_09622802241262521 - Supplemental material for Analyzing heterogeneity in biomarker discriminative performance through partial time-dependent receiver operating characteristic curve modeling [file sj-pdf-1-smm-10.1177_09622802241262521.pdf]

Supplementary materials for "Analyzing Heterogeneity in Biomarker Discriminative Performance through Partial Time-Dependent Receiver Operating Characteristic Curve Modeling"

Xinyang Jiang[1] Wen Li[2] Kang Wang[3] Ruosha Li[1]
Jing Ning †[3], for the Alzheimer's Disease Neuroimaging Initiative*

[1]Department of Biostatistics and Data Science, The University of Texas Health Science Center at Houston, TX, USA
[2]Department of Internal Medicine, The University of Texas Health Science Center at Houston McGovern Medical School, TX, USA
[3]Department of Biostatistics, The University of Texas MD Anderson Cancer Center, 1400 Pressler St., Houston, TX, USA

S1 Proof of Theorem 1

We impose the following regularity conditions to establish the asymptotic properties of the proposed method:

(A1) $P(\tilde{T} < C) > 0$.

(A2) The matrices $\mathbf{\Sigma}_1$ and $\mathbf{\Sigma}_2$ defined in Theorem 1 are both finite and positive-definite.

(A3) The covariate vector $\mathbf{X}$ lies within a bounded compact set $\mathcal{X}$ in $\mathbb{R}^p$, and it maintains full rank.

(A4) $PAUC^*(t; u, \mathbf{x}, \boldsymbol{\theta})$ is bounded away from 0 and 1 in a small neighborhood of $\boldsymbol{\theta}_0$, and is continuous and twice differentiable with respect to $\boldsymbol{\theta}$.

(A5) When $n \to \infty$, the bandwidth h satisfies (i) $0 \leq h \to 0$, (ii) $nh/\log n \to \infty$, (iii) $n^{1/4}h \to 0$, and (iv) $(n/\log n)^{1-2/\lambda}h \to \infty$ (Hu T, et al. 2019).

(A6) The kernel function is bounded and possesses bounded variation.

We begin by establishing the consistency of $\hat{\boldsymbol{\theta}}$. For any $\delta_{\boldsymbol{\theta}} > 0$, the intersection of the parameter space of $\boldsymbol{\theta}$ and a $\delta_{\boldsymbol{\theta}}$-neighborhood of $\boldsymbol{\theta}_0$ is closed. As a result, a local maximum of the likelihood function $L(\boldsymbol{\theta})$ exists within this set. Given the assumption (A4), the negative-definite second-order derivative of $L(\boldsymbol{\theta})$ with respect to $\boldsymbol{\theta}$ is well defined. By the Taylor expansion of $L(\boldsymbol{\theta})$ around $\hat{\boldsymbol{\theta}}$, it can be shown that as $n \to \infty$, $L(\boldsymbol{\theta}) < L(\boldsymbol{\theta}_0)$ for all $\boldsymbol{\theta}$ lying within the parameter space and at a distance of $\delta_{\boldsymbol{\theta}}$ from $\boldsymbol{\theta}_0$, with a probability converging to 1 (Chen et al. 2005). By conditions (A5) and (A6), and similar arguments in Hu T, et al. (2019), we can show that the impact from kernel smoothing is negligible. Consequently, the maximum is located within the parameter space of $\boldsymbol{\theta}$, and its distance from $\boldsymbol{\theta}_0$ is less than $\delta_{\boldsymbol{\theta}}$ with a probability tending to 1. Thus, the consistency of $\hat{\boldsymbol{\theta}}$ is established.

†Correspondence: Department of Biostatistics, The University of Texas MD Anderson Cancer Center, 1400 Pressler St., Houston, TX 77030, USA. Email: jning@mdanderson.org

*Data used in preparation of this article were obtained from the Alzheimer's Disease Neuroimaging Initiative (ADNI) database (adni.loni.usc.edu). As such, the investigators within the ADNI contributed to the design and implementation of ADNI and/or provided data but did not participate in analysis or writing of this report. A complete listing of ADNI investigators can be found at: `http://adni.loni.usc.edu/wp-content/uploads/how_to_apply/ADNI_Acknowledgement_List.pdf`

To prove the asymptotic normality, we consider the Taylor expansion of $S(\boldsymbol{\theta})$ around $\boldsymbol{\theta}_0$ as follows:

$$0 = \sum_i \sum_j S_{ij}(\hat{\boldsymbol{\theta}}) \approx \sum_i \sum_j S_{ij}(\boldsymbol{\theta}_0) + (\hat{\boldsymbol{\theta}} - \boldsymbol{\theta}_0) \sum_i \sum_j \nabla_{\boldsymbol{\theta}} S_{ij}(\boldsymbol{\theta}_0),$$

where S_{ij} represents the score equation defined in the main text. This leads to the following conclusion:

$$\sqrt{n}(\hat{\boldsymbol{\theta}} - \boldsymbol{\theta}_0) = \mathbf{A}_n^{-1} n^{1/2} \mathbf{B}_n + O_p(n^{-1/2}),$$

where $\mathbf{A}_n = -\sum_i \sum_j \nabla_{\boldsymbol{\theta}} S_{ij}(\boldsymbol{\theta}_0)/n^2$ and $\mathbf{B}_n = \sum_i \sum_j S_{ij}(\boldsymbol{\theta}_0)/n^2$. Utilizing the law of large numbers, it follows that $\mathbf{A}_n$ converges to Σ_1 with a probability of 1 as $n \to \infty$. Define $G_{ij}(\boldsymbol{\theta}) = \{S_{ij}(\boldsymbol{\theta}) + S_{ji}(\boldsymbol{\theta})\}/2$, which implies that $\mathbf{B}_n = \sum_{i<j} G_{ij}(\boldsymbol{\theta}_0)/\{n(n-1)/2\}$, resulting in $\mathbf{B}_n$ being a U-statistic of degree 2. By applying Theorem 6.1.4 in Lehmann EL (2004), it can be established that $n^{1/2} B_n \to N(0, \Sigma_2)$ as $n \to \infty$. Under the regularity conditions, the kernel smoothing does not introduce additional variation to the asymptotic variance of the estimators. By invoking the Slutsky's Theorem, the asymptotic distribution of $\hat{\boldsymbol{\theta}}$ can be obtained.

S2 Additional Simulation Results

The simulation results referenced in the main body of this paper are presented in Table S1-S3 and Figure S1-S4.

Table S1: Simulation results in Scenario 1

		Cen =0%							
		N=500				N=1000			
u	TRUE	BIAS	SD	SE	CP	BIAS	SD	SE	CP
0.2	0.607	-0.032	0.201	0.200	0.943	-0.021	0.140	0.144	0.947
0.4	0.567	-0.013	0.173	0.165	0.935	-0.011	0.122	0.117	0.940
0.6	0.535	-0.011	0.158	0.146	0.924	-0.008	0.112	0.104	0.931
0.8	0.501	-0.007	0.146	0.135	0.929	-0.006	0.104	0.096	0.935
1.0	0.464	0.003	0.136	0.134	0.944	0.000	0.096	0.095	0.951
		Cen =15%							
0.2	0.622	-0.028	0.215	0.208	0.938	-0.021	0.140	0.144	0.947
0.4	0.582	-0.009	0.184	0.173	0.935	-0.011	0.122	0.117	0.940
0.6	0.535	-0.011	0.158	0.146	0.924	-0.008	0.112	0.104	0.931
0.8	0.515	-0.006	0.155	0.143	0.935	-0.006	0.104	0.096	0.935
1.0	0.478	0.004	0.144	0.142	0.950	0.000	0.096	0.095	0.951
		Cen =30%							
0.2	0.641	-0.023	0.227	0.220	0.940	-0.012	0.163	0.159	0.943
0.4	0.598	-0.003	0.198	0.184	0.925	-0.001	0.140	0.131	0.931
0.6	0.535	-0.011	0.158	0.146	0.924	-0.008	0.112	0.104	0.931
0.8	0.530	0.001	0.171	0.154	0.919	0.003	0.118	0.109	0.928
1.0	0.492	0.010	0.159	0.152	0.941	0.008	0.110	0.108	0.943

Note: SD: empirical standard deviations; SE: average of estimated standard errors; CP: coverage probabilities.

Table S2: Simulation results in Scenario 2.

		Cen =0%							
		N=500				N=1000			
u	TRUE	BIAS	SD	SE	CP	BIAS	SD	SE	CP
0.2	0.599	-0.116	0.307	0.302	0.928	-0.071	0.217	0.219	0.946
0.4	0.558	-0.074	0.272	0.255	0.923	-0.043	0.194	0.182	0.932
0.6	0.526	-0.060	0.256	0.229	0.924	-0.033	0.182	0.164	0.917
0.8	0.494	-0.029	0.242	0.212	0.919	-0.016	0.171	0.151	0.910
1.0	0.459	-0.008	0.227	0.214	0.938	-0.003	0.160	0.152	0.935
		Cen =15%							
0.2	0.614	-0.119	0.337	0.318	0.912	-0.072	0.233	0.231	0.930
0.4	0.579	-0.087	0.296	0.271	0.909	-0.053	0.202	0.194	0.925
0.6	0.549	-0.076	0.276	0.245	0.900	-0.036	0.191	0.174	0.919
0.8	0.516	-0.066	0.259	0.227	0.908	-0.029	0.179	0.163	0.922
1.0	0.481	-0.025	0.248	0.228	0.924	-0.016	0.166	0.162	0.937
		Cen =30%							
0.2	0.645	-0.101	0.353	0.331	0.923	-0.073	0.253	0.242	0.932
0.4	0.606	-0.068	0.308	0.289	0.924	-0.049	0.221	0.208	0.921
0.6	0.575	-0.052	0.286	0.264	0.930	-0.042	0.209	0.189	0.918
0.8	0.540	-0.043	0.269	0.248	0.929	-0.036	0.195	0.177	0.922
1.0	0.504	-0.024	0.253	0.247	0.951	-0.024	0.183	0.176	0.932

Note: SD: empirical standard deviations; SE: average of estimated standard errors; CP: coverage probabilities.

Table S3: Simulation results in Scenario 3.

			Cen =0%							
			N=500				N=1000			
u	Coef.	TRUE	BIAS	SD	SE	CP	BIAS	SD	SE	CP
0.2	β_d	0.609	-0.100	0.200	0.188	0.896	-0.070	0.140	0.138	0.914
	β_c	0.598	-0.189	0.301	0.289	0.890	-0.118	0.217	0.213	0.907
0.4	β_d	0.568	-0.056	0.173	0.160	0.906	-0.039	0.123	0.115	0.910
	β_c	0.559	-0.129	0.270	0.250	0.903	-0.079	0.195	0.180	0.905
0.6	β_d	0.535	-0.036	0.160	0.144	0.910	-0.029	0.115	0.103	0.905
	β_c	0.529	-0.107	0.254	0.226	0.906	-0.047	0.186	0.162	0.898
0.8	β_d	0.500	-0.028	0.150	0.134	0.913	-0.019	0.107	0.095	0.915
	β_c	0.497	-0.061	0.242	0.211	0.907	-0.037	0.173	0.151	0.897
1	β_d	0.465	0.001	0.144	0.134	0.924	-0.002	0.100	0.095	0.942
	β_c	0.464	-0.004	0.233	0.214	0.925	-0.019	0.162	0.152	0.929
			Cen =15%							
0.2	β_d	0.620	-0.087	0.210	0.196	0.910	-0.059	0.149	0.144	0.923
	β_c	0.619	-0.194	0.333	0.305	0.876	-0.124	0.236	0.224	0.904
0.4	β_d	0.582	-0.047	0.185	0.168	0.923	-0.035	0.132	0.121	0.914
	β_c	0.582	-0.145	0.294	0.266	0.885	-0.090	0.204	0.192	0.900
0.6	β_d	0.550	-0.037	0.170	0.151	0.914	-0.024	0.122	0.109	0.906
	β_c	0.555	-0.097	0.282	0.242	0.889	-0.078	0.191	0.174	0.902
0.8	β_d	0.516	-0.025	0.159	0.142	0.917	-0.020	0.114	0.101	0.911
	β_c	0.521	-0.082	0.266	0.226	0.884	-0.049	0.181	0.163	0.908
1	β_d	0.480	0.001	0.149	0.142	0.935	-0.003	0.107	0.101	0.930
	β_c	0.486	-0.053	0.252	0.227	0.913	-0.031	0.169	0.163	0.935
			Cen =30%							
0.2	β_d	0.642	-0.079	0.229	0.207	0.913	-0.054	0.162	0.153	0.923
	β_c	0.649	-0.189	0.357	0.328	0.884	-0.130	0.254	0.242	0.899
0.4	β_d	0.602	-0.038	0.203	0.180	0.910	-0.027	0.141	0.129	0.918
	β_c	0.609	-0.148	0.313	0.288	0.900	-0.097	0.222	0.208	0.903
0.6	β_d	0.569	-0.027	0.189	0.163	0.896	-0.019	0.131	0.117	0.910
	β_c	0.579	-0.091	0.297	0.263	0.906	-0.067	0.213	0.189	0.901
0.8	β_d	0.534	-0.015	0.177	0.154	0.905	-0.011	0.122	0.109	0.915
	β_c	0.544	-0.077	0.280	0.248	0.905	-0.059	0.200	0.178	0.908
1	β_d	0.498	0.009	0.166	0.153	0.916	0.004	0.114	0.109	0.923
	β_c	0.508	-0.051	0.265	0.247	0.934	-0.041	0.188	0.177	0.930

Note: SD: empirical standard deviations; SE: average of estimated standard errors; CP: coverage probabilities.

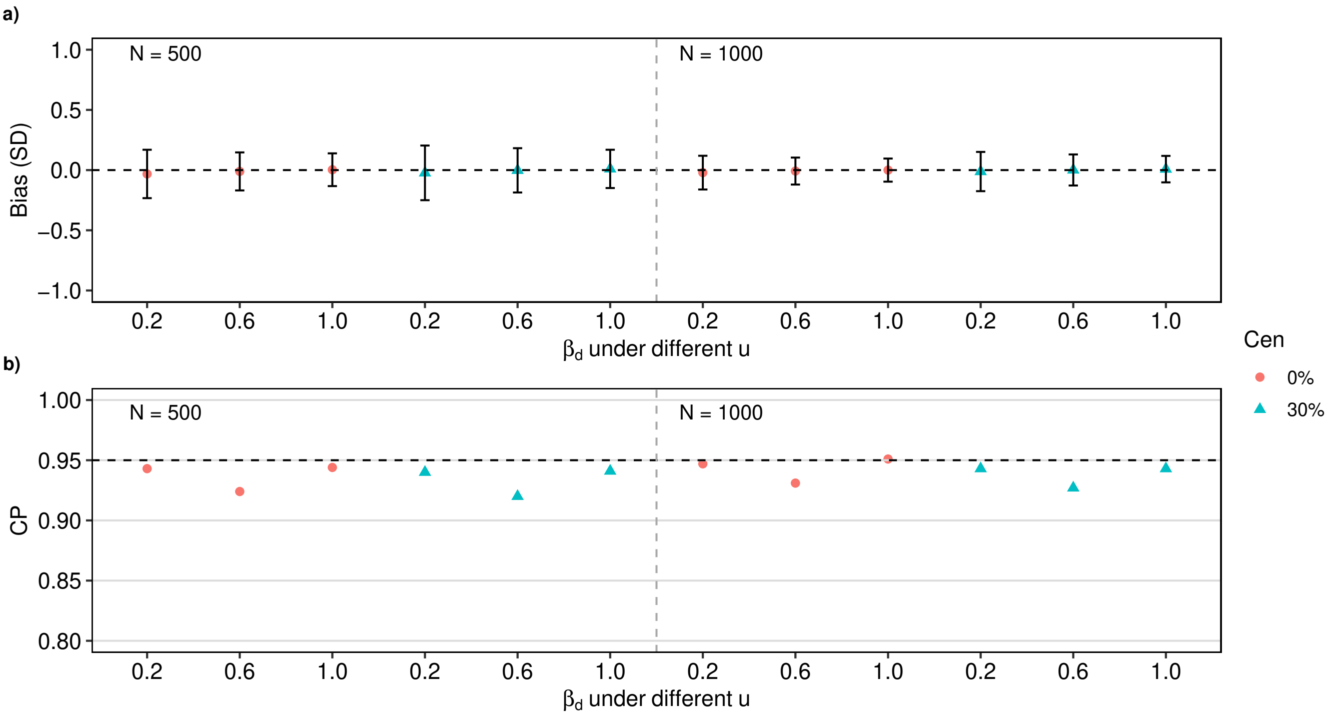

Figure S1: Simulation Results of a) Biases, Empirical Standard Deviations and b) Empirical Coverage Probabilities in Scenario 1

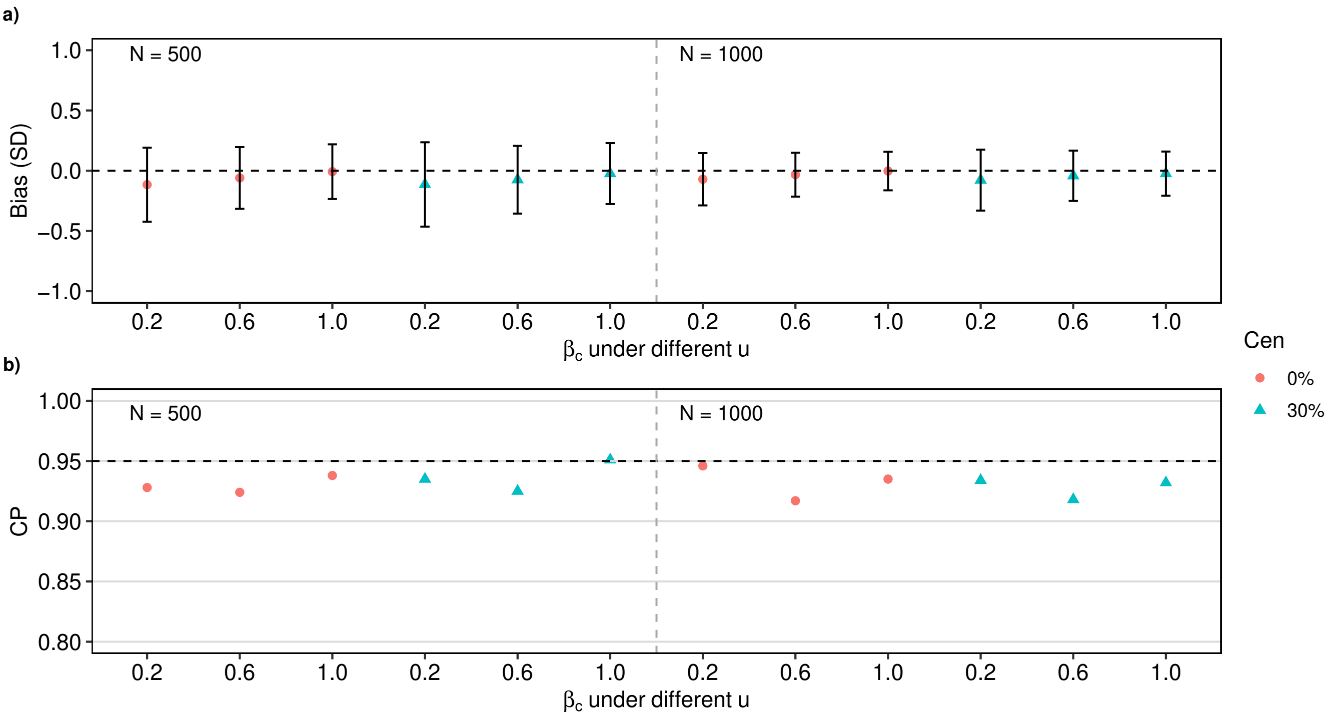

Figure S2: Simulation Results of a) Biases, Empirical Standard Deviations and b) Empirical Coverage Probabilities in Scenario 1

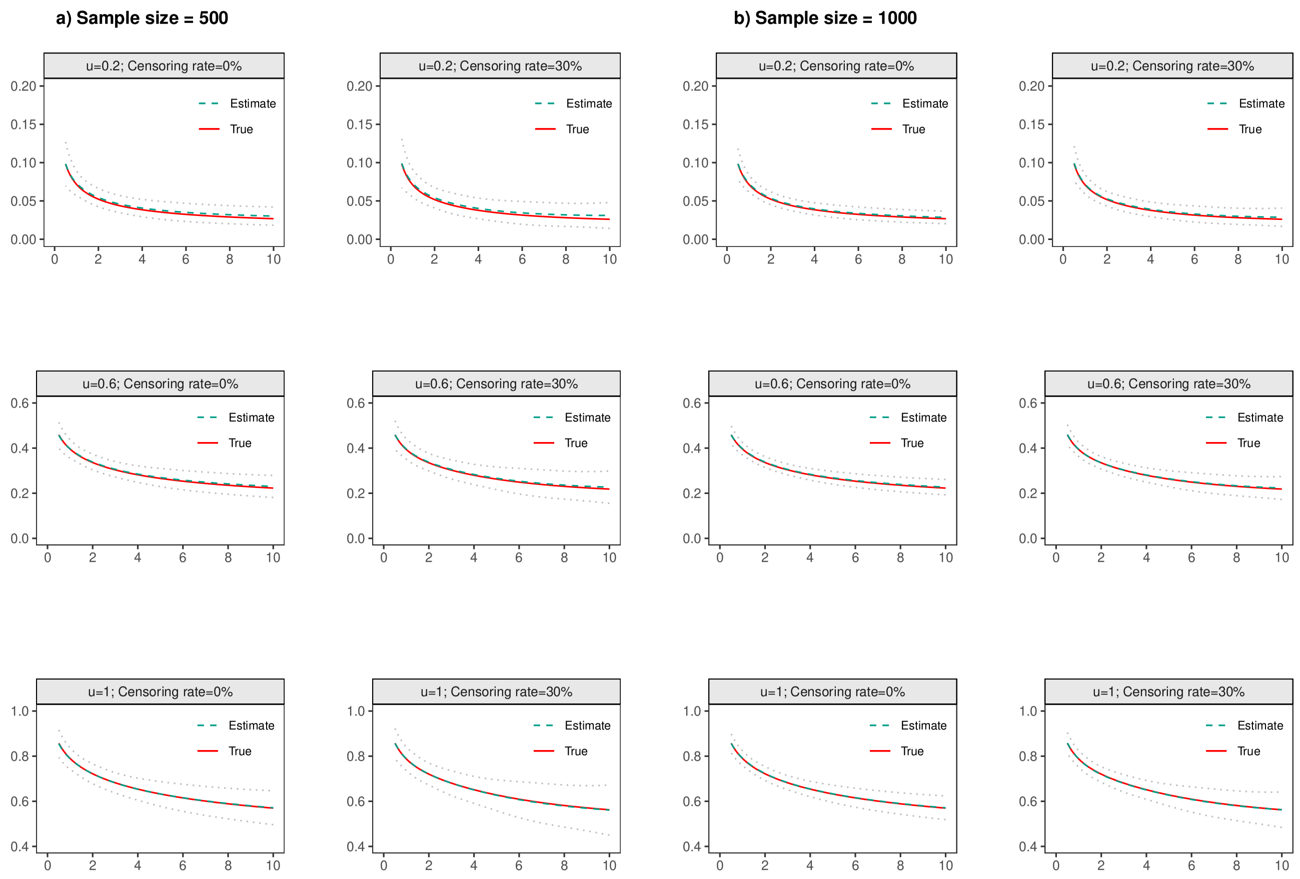

Figure S3: Estimated baseline $PAUC_0(t, \boldsymbol{\alpha})$ with 95% Confidence Intervals in Scenario 1

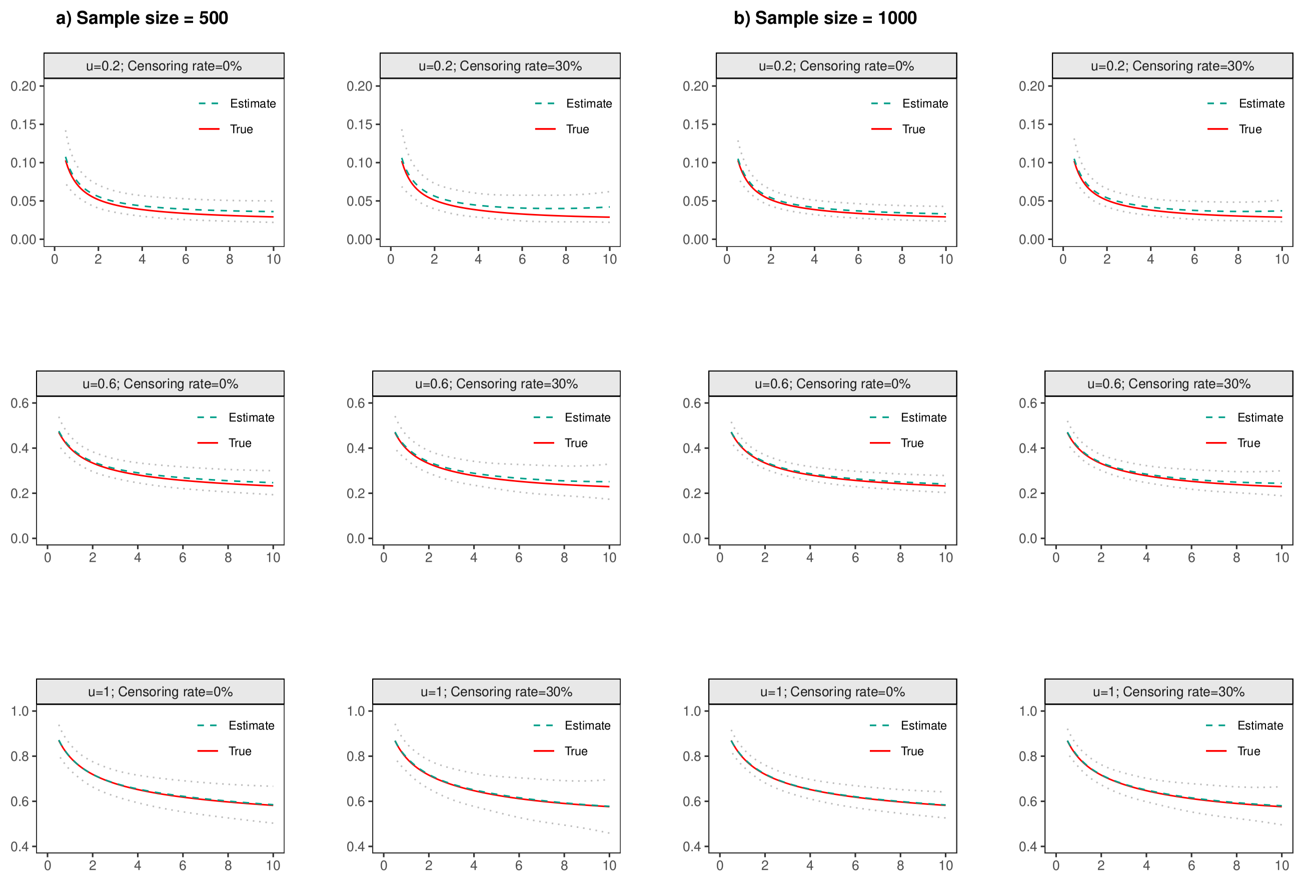

Figure S4: Estimated baseline $PAUC_0(t, \boldsymbol{\alpha})$ with 95% Confidence Intervals in Scenario 2

We acknowledge the inherent risk of over-fitting associated with the flexibility of fractional polynomials, particularly in scenarios with small sample sizes. In our case, the full model incorporates seven fractional polynomial terms, which render reliable performance for sample sizes of either 500 or 1000 in simulation studies and our data application. To address this concern, we conducted a sensitivity study to compare the performance of the full model against models with five or three polynomial terms under the following model specifications respectively:

$$logit\{PAUC^*(t; x_d, x_c)\} = \alpha_0 + \alpha_2 t^{-1} + \alpha_3 t^{-1/2} + \alpha_4 \log(t) + \alpha_5 t^{1/2} + \alpha_6 t + \beta_d x_d + \beta_c x_c.$$

and

$$logit\{PAUC^*(t; x_d, x_c)\} = \alpha_0 + \alpha_5 t^{1/2} + \alpha_6 t + \alpha_7 t^2 + \beta_d x_d + \beta_c x_c.$$

We initially fitted the model with only 3 fractional polynomials to the large dataset to estimate the true values and then fitted the models with 3, 5, and 7 fractional polynomials to examine the impact of a more flexible specification on model performance. The simulation results, as displayed in Table S4, confirmed that both the 5-polynomial and 7-polynomial models produced covariate effects on the PAUC that are nearly indistinguishable from those of the 3-polynomial model. As expected, the biases of the 5-polynomial and 7-polynomial models were slightly larger than those of the 3-polynomial model, as the true values were approximated by the model with 3 polynomials. This observation suggests that the proposed model demonstrates robust performance in analyzing the heterogeneity in biomarker performance concerning the number of polynomials included, particularly in estimating the covariate effects on PAUC. Furthermore, our analysis indicates that either the 3-polynomial model or these alternative specifications suffice in our context, as we anticipate no dramatic fluctuations in the time-dependent PAUC value over time. In the revised version, we have incorporated the sensitivity analysis.

Table S4: Simulation Results for the Sensitivity Study

Number of fraction polynomial terms	Coef.	True	Bias	SD	SE	CP
3	β_d	0.488	-0.005	0.104	0.102	0.941
	β_c	0.483	-0.004	0.171	0.164	0.942
5	β_d	0.488	-0.015	0.101	0.101	0.954
	β_c	0.483	-0.042	0.165	0.161	0.935
7	β_d	0.488	-0.015	0.102	0.101	0.951
	β_c	0.483	-0.044	0.164	0.161	0.935

Additionally, we conducted a simulation study under settings similar to Scenario 3, but with a change in the distribution of censoring times. In this additional simulation study, we generated censoring times from the Weibull distribution derived from the ADNI data. The event time $\widetilde{T}$ was generated from a log-normal distribution, adjusted by a constant ratio of 0.08 to achieve a censoring rate of 70%. The simulation results remained consistent across scenarios with lower censoring rates and a uniform distribution of censoring times. The biases across these settings were small, although biases tended to increase slightly with smaller effective sample sizes (i.e., a lower u). As a result, the coverage probabilities (CPs) exhibited a slight decline below the nominal value as u decreased.

To further investigate the performance of our proposed method under scenarios involving interaction terms, we conducted an additional simulation study by incorporating interaction terms on μ_Y and $\mu_{log(T)}$. Specifically, we generated two independent covariates: $X_d \sim Bernoulli(0.5)$ and $X_c \sim Uniform(0, 1)$. The mean of the biomarker was set as $\mu_Y = -X_d - X_c - X_d X_c - 1$ while the mean of the log-transformed time was set as $\mu_{log(T)} = X_d + X_c + X_d X_c + 1$. We fitted the following model:

$$logit\{PAUC^*(t; x_d, x_c)\} = \alpha_0 + \alpha_1 t^{-2} + \alpha_2 t^{-1} + \alpha_3 t^{-1/2} + \alpha_4 \log(t) + \alpha_5 t^{1/2} + \alpha_6 t + \alpha_7 t^2 + \beta_1 x_d + \beta_2 x_c + \beta_3 x_d x_c.$$

Table S5: Simulation Results under Scenario with Weibull Censoring Distribution, Sample Size 1000 and Censoring Rate 70%

u	Parameter	TRUE	BIAS	SD	SE	CP
0.2	β_d	0.757	-0.051	0.233	0.209	0.909
	β_c	0.751	-0.054	0.377	0.329	0.909
0.4	β_d	0.726	-0.027	0.211	0.192	0.916
	β_c	0.713	-0.020	0.333	0.301	0.921
0.6	β_d	0.694	-0.016	0.199	0.181	0.923
	β_c	0.679	-0.009	0.313	0.283	0.933
0.8	β_d	0.656	-0.009	0.188	0.173	0.927
	β_c	0.640	-0.003	0.296	0.270	0.934
1.0	β_d	0.618	0.005	0.179	0.169	0.934
	β_c	0.603	0.011	0.279	0.266	0.945

The simulation results, as summarized in Table S6, demonstrate the model's capability to accommodate interaction terms. The small biases and close alignment between the standard deviation (SD) and the standard error (SE) across all coefficients, coupled with coverage probabilities (CP) near the nominal level, show the model's effectiveness in incorporating interaction effects.

Table S6: Simulation Results with an Interaction Term (β_3) and a Sample Size of 1000

**Parameter**	**TRUE**	**BIAS**	**SD**	**SE**	**CP**
β_1	0.512	0.029	0.201	0.183	0.921
β_2	0.515	-0.048	0.228	0.216	0.927
β_3	0.380	-0.063	0.355	0.327	0.920

S3 Additional Data Application Results

In this section, we present descriptive analysis and regression analysis results for biomarkers, Aβ42 and p-tau, as referenced in the main body of this paper.

Table S7: Descriptive Analysis for Baseline Patient Characteristics and CSF Biomarkers

**Variable**	**Mean**	**Standard Deviation**	**Median**	**Interquartile Range**
Age	73	7	73	68 - 78
Aβ42	1052.2	454.7	955.7	664.3 - 1533.0
p-tau	25.5	13.2	21.9	16.3 - 31.3
t-tau	268.0	118.1	242.3	185.4 - 322.8

Table S8: Estimated covariate effects on the PAUC of biomarker Aβ42 using the ADNI data

u	Covariate	Estimate	SE	Wald	P-value
0.4	Gender (Female)	-0.729	0.733	-0.995	0.320
	Age	0.191	0.354	0.540	0.589
	Age^2	0.898	0.372	2.415	0.016
0.6	Gender (Female)	-1.180	0.549	-2.149	0.032
	Age	0.266	0.299	0.889	0.374
	Age^2	0.583	0.325	1.792	0.073
0.8	Gender (Female)	-0.729	0.293	-2.489	0.013
	Age	0.255	0.224	1.138	0.255
	Age^2	0.378	0.228	1.661	0.097
1	Gender (Female)	-0.230	0.167	-1.373	0.170
	Age	0.347	0.152	2.283	0.022
	Age^2	0.043	0.184	0.235	0.814

Table S9: Estimated covariate effects on the PAUC of biomarker p-tau using the ADNI data

u	Covariate	Estimate	SE	Wald	P-value
0.2	Gender (Female)	0.469	0.250	1.878	0.060
	Age	-0.029	0.227	-0.128	0.898
	Age^2	-0.174	0.250	-0.695	0.487
0.4	Gender (Female)	0.380	0.201	1.888	0.059
	Age	-0.199	0.189	-1.051	0.293
	Age^2	-0.272	0.201	-1.354	0.176
0.6	Gender (Female)	0.421	0.197	2.144	0.032
	Age	-0.221	0.179	-1.234	0.217
	Age^2	-0.288	0.187	-1.541	0.123
0.8	Gender (Female)	0.437	0.194	2.255	0.024
	Age	-0.199	0.172	-1.158	0.247
	Age^2	-0.338	0.174	-1.941	0.052
1	Gender (Female)	0.472	0.186	2.532	0.011
	Age	-0.250	0.164	-1.519	0.129
	Age^2	-0.334	0.164	-2.032	0.042

References

Hu, T., Nan, B., & Lin, X. (2019). Proportional cross-ratio model. Lifetime data analysis, 25, 480-506.

Chen, Y., Ning, J., & Cai, C. (2015). Regression analysis of longitudinal data with irregular and informative observation times. Biostatistics, 16(4), 727-739.

Lehmann, E. L. (2004). Elements of large-sample theory (3rd ed.). New York, NY: Springer New York.
